# The small GTPases FoRab5, FoRab7, and FoRab8 regulate vesicle transport to modulate vegetative development and pathogenicity in *Fusarium oxysporum* f. sp. *conglutinans*

**DOI:** 10.3389/fmicb.2025.1514092

**Published:** 2025-01-29

**Authors:** Xiangyu Tan, Lin Chen, Ying Chen, Yuxin Li, Lihan Lu, Erfeng Li

**Affiliations:** ^1^Horticulture and Landscape College, Tianjin Agricultural University, Tianjin, China; ^2^Comprehensive Utilization of Edible and Medicinal Plant Resources Engineering Technology Research Center, Zhengzhou Key Laboratory of Synthetic Biology or Natural Products, Zhengzhou Key Laboratory of Medicinal Resources Research, Huanghe Science and Technology College, Zhengzhou, China

**Keywords:** *Fusarium oxysporum*, Rab GTPases, gene knockout, vesicle transport, pathogenicity

## Abstract

Rab GTPases play a crucial role in facilitating the transportation of vesicles during the process of fungal biogenesis. Currently, there is limited understanding regarding the specific biological functions of Rab small GTPase elements within *Fusarium oxysporum*. In this study, we examined the three proteins FoRab5, FoRab7, and FoRab8 of Foc, which exhibit homology to the Rab protein family found in *Saccharomyces cerevisiae*. In addition, we also employed a PEG-mediated homologous recombination approach to create deletion mutants and complementary strains for the *FoRab5*, *FoRab7*, and *FoRab8* genes, thereby facilitating a comprehensive investigation into the functional roles of these genes. FoRab5 was localized on vesicles of various sizes within the cell. Compared to the wild-type strain, the growth rate of the mutant *ΔFoRab5* strain decreased, the aerial hyphae decreased, the sporulation decreased, and the spore germination rate decreased. The sensitivity to cell membrane stress, cell wall stress, and endoplasmic reticulum stress increased, the activity of laccase and glucoamylase decreased significantly, and the pathogenicity to cabbage seedlings decreased. FoRab7 was localized on the vacuolar membrane. Compared to the wild type, the growth rate of the mutant *ΔFoRab7* strain decreased, the bacteria produced a large amount of pigment deposition, the aerial hyphae decreased significantly, the hyphal branches increased, and the mutant almost lost the ability to produce spores. The sensitivity to osmotic stress, cell membrane stress, cell wall stress, metal ion stress, and endoplasmic reticulum stress was enhanced, and the vacuole was fragmented. Laccase and glucoamylase activities decreased in a significant manner. Moreover, there was a decrease in the pathogenicity of cabbage seedlings. FoRab8 was localized at the tip of the mycelium. Compared to the wild type, the growth rate of the mutant *ΔFoRab8* strain decreased, the sporulation decreased, and the sensitivity of the mutant to osmotic stress and endoplasmic reticulum pressure increased. There was a significant decrease in the activity of laccase, glucoamylase, and cellulase. A reduction in the pathogenicity to cabbage seedlings occurred. In summary, these results indicate that members of the Rab family proteins FoRab5, FoRab7, and FoRab8 regulate a series of processes such as growth, sporulation, pathogenicity, and ectoenzyme secretion in Foc.

## 1 Introduction

Cabbage wilt is a fungal soil-borne disease caused by *Fusarium oxysporum* f. sp. *conglutinans*. Worldwide, cabbage wilt disease was first discovered in Hudson Valley, New York, USA, in 1895. This disease was first discovered in China in Yanqing, Beijing, in 2001 (Geng et al., 2009; Liu et al., 2019). It has gradually become a major obstacle to the production and cultivation of cruciferous vegetables. Cabbage wilt is a soil-borne disease that can occur at both the seedling and mature plant stages. The pathogen infects the vascular bundle system of the host plant through conidia (Sherf and Macnab, 1986). It causes yellowing and wilting of the lower older leaves first and then gradually spreads to the upper leaves (Lv et al., 2013). This leads to the obstruction and browning of the plant’s vascular bundle tissues, ultimately resulting in the wilting and even death of the entire plant (Pu et al., 2012). Vesicular transport is an extremely complex dynamic process that primarily includes the steps of vesicle budding, transport, tethering, docking, priming, triggering, and fusion (Vetter and Wittinghofer, 2001). In the final fusion, the vesicle must accurately identify and recognize the correct target membrane with high precision (Yang et al., 2024). Vesicular transport includes endocytosis and exocytosis. Exocytosis is the process by which cells form vesicles through the plasma membrane to transport extracellular material needed by the cell to maintain life activities. Depending on the status of transcytosis, endocytosis is divided into pinocytosis and phagocytosis (Mani and Singh, 2023). Exocytosis refers to the process by which biologically large molecules and metabolites synthesized within the cell are fused with the plasma membrane in the form of vesicles, and their contents are secreted to the cell surface or outside the cell (Zhang et al., 2024). Proteins that have completed translation need to be transported to specific parts of the cell to perform their functions, thus participating in the normal life activities of the cell. This process is mediated and completed by exocytosis.

Eukaryotic cells rely on the intricate mechanism of vesicle trafficking to ensure that different proteins are transported to their correct locations, thus enabling them to perform their biological functions. Vesicle trafficking requires the participation of a large family of proteins, mainly including SNARE, Arf, SM, and Rab. These protein families are all highly conserved (Hashizume et al., 2009). Rab is a small GTP-binding protein that plays an important regulatory role after vesicle budding. It can regulate the transport of vesicles along the protein fiber skeleton in cells, modulate the recognition and anchoring of vesicles to specific receptors on the membrane of organelles or cell membranes, and regulate the fusion process between vesicles and the membrane of organelles or cell membranes (Mizuno-Yamasaki et al., 2012). Rab family proteins possess intrinsic GTPase activity, serving as molecular switches (Yang, 2002). By cycling between inactive (GDP-bound) and active (GTP-bound) states, Rab GTPases coordinate with their numerous effector proteins to enable precise spatial targeting of secretory vesicles (Kabcenell et al., 1990). The first Rab proteins to be discovered and identified are Sec4 and Ypt1 proteins in *S. cerevisiae*, which participate in endocytosis and exocytosis pathways (Salminen and Novick, 1987; Segev et al., 1988). In the field of fungi, Rab family proteins have been extensively studied in yeast. In recent years, researchers have begun to explore the functions of Rab family proteins in plant pathogenic fungi and have made a series of advances. Rab proteins are highly conserved in evolution, consisting of several important domains: the G domain (G1–G5), RabF motifs (RabF1–RabF5), RabSF motifs (RabSF1–RabSF4), and the C-terminal cysteine motif (C motif) (Dumas et al., 1999). In addition, the C-terminus of Rab proteins contains a membrane-targeting signal. When the cysteine at the C-terminus is modified by prenylation, Rab proteins are anchored to the membrane through their C-terminal end.

Research reports have shown that in various plant pathogenic fungi, Rab family proteins are closely related to mycelial morphology and growth rate, conidial production and spore germination rate, pathogenicity, toxin production, and other aspects. PlRAB5A of *Peronophythora litchii* is localized on the vesicle membrane and primary endosome, potentially performing functions throughout the entire life cycle of *P. litchii*. Deletion mutants exhibit significantly reduced growth rates, irregular enlargement of hyphae with increased septation, decreased release of zoospores, and significantly reduced pathogenicity (Yang, 2020). MoYpt7 of *Magnaporthe oryzae* is mainly localized on the vacuolar membrane. After deleting the *MoYPT7* gene, the mutant exhibits malformed conidia, fails to form appressoria, lacks pathogenicity and displays autophagy damage, disruption of cell wall integrity, and increased sensitivity to calcium and heavy metal stress (Liu et al., 2015). *SrgA* of *Aspergillus fumigatus* is localized at the hyphal tip and mature conidiophores. The *SrgA* mutant exhibits impaired vegetative growth, abnormal conidial morphology, and high sensitivity to vesicular transport inhibition (Powers-Fletcher et al., 2013). The specific mechanism of the Rab protein family related to vesicle transport is still in the research stage. It has been found that members of the Rab protein family can play an important role in various plant pathogenic fungi. However, there is still a lack of research reports on the Rab family-related proteins in Foc. In this study, a variety of molecular biology methods were used to clarify the subcellular localization of the protein and study the differences between the mutant, wild-type, and complement strains in biological characteristics, related functional phenotypes, and infected hosts. The purpose of this study was to systematically elucidate the biological functions of FoRab5, FoRab7, and FoRab8 in the Rab family proteins of Foc and their effects on pathogenicity and clarify their regulation of Foc growth and development and parasitic pathogenesis. It lays a foundation for the subsequent functional study of Rab proteins.

## 2 Materials and methods

### 2.1 Fungal strains, culture media, and growth conditions

Foc strain R1 was used as the wild-type strain in this study (Li et al., 2015a). The Foc wild-type isolate was routinely grown on potato dextrose agar (PDA) at 28°C. Deletion mutants (*ΔFoRab5*, *ΔFoRab7*, and *ΔFoRab8*) and complementation mutants (*ΔFoRab5*-C, *ΔFoRab7*-C, and *ΔFoRab8*-C) were maintained on PDA containing 80 μg/mL hygromycin B or 160 μg/mL neomycin under the same conditions.

### 2.2 Construction of homolog replacement arms for the target genes and their complement vector

To dissect the effects of *FoRab5*, *FoRab7*, and *FoRab8* gene deletions, the homologous replacement arms of the three genes were constructed, respectively. The upstream of the start codon and the downstream of the stop codon of the three target genes were amplified from the genomic DNA of Foc with the corresponding primers. The three target genes were replaced with the 1,376 bp fragment that encoded hygromycin B phosphotransferase (*hygR*) as a selectable marker. Then, the upstream and downstream of the three target genes were ligated with *hygR* and amplified to obtain the homologous replacement arms for fungal transformation.

To achieve gene complementation, the complementary plasmids of three target genes were constructed and transferred into the corresponding mutant strains for PCR verification. To construct the pRGTN-*FoRab5*, pRGTN-*FoRab7*, and pRGTN-*FoRab8* plasmids, the coding regions of *FoRab5*, *FoRab7*, and *FoRab8* were amplified from the cDNA of Foc. The resulting amplicon was cloned into *Eco*RI/*Hind*III-digested pRGTN to produce complementary plasmids. The plasmids were then sequenced and introduced into the *ΔFoRab5*, *ΔFoRab7*, and *ΔFoRab8* strains for genetic complementation and localization studies.

### 2.3 Transformation procedures mediated by PEG-CaCl_2_

To prepare protoplasts, the tender mycelium germinated for 16 h was collected into a micro-centrifuge tube and treated with Driselase^-^™ for 3–4 h (Li et al., 2011). The protoplasts were then transferred to micro-centrifuge tubes at 100 μL/tube for use. Transformation components were added to 100 μL of protoplast suspension and mixed gently with a pipette tip (Turgeon et al., 1987; Talbot et al., 1993). Next, 160 μL of PEG solution was dropped into the DNA-protoplast suspension. The protoplast suspension was then mixed with the regeneration medium and poured onto the regeneration medium. After 16 h, the transformants were screened by covering a regeneration medium containing 80 μg/mL hygromycin B or 160 μg/mL neomycin (Li et al., 2016).

Deletion mutants and complementation transformants were selected and maintained on PDA medium containing 80 μg/mL hygromycin B or 160 μg/mL neomycin. The correct deletion mutants (*ΔFoRab5*, *ΔFoRab7*, and *ΔFoRab8*) and complementation mutants (*ΔFoRab5*-C, *ΔFoRab7*-C, and *ΔFoRab8*-C) were confirmed by PCR.

### 2.4 Analysis of subcellular localization by GFP tagging

Initially, the WoLF PSORT online platform^1^ was utilized to predict the subcellular localization of FoRab5, FoRab7, and FoRab8. To obtain strains suitable for observing subcellular localization, the coding regions of the *FoRab5*, *FoRab7*, and *FoRab8* genes were first amplified and subsequently inserted into a vector containing green fluorescent protein. The expression of the green fluorescent protein was regulated using the ribosomal protein gene promoter RP27. Positive clones were then selected, after which the recombinant vectors were introduced into mutant strains (*ΔFoRab5*, *ΔFoRab7*, and *ΔFoRab8*) for identification.

As mentioned above, three strains (GFP-FoRab5, GFP-FoRab7, and GFP-FoRab8) for subcellular localization observation were obtained. Fresh hyphae were collected from the PDB culture medium after 24 h of shaking for live cell imaging. Live cell imaging was conducted using a laser scanning confocal microscopy system. GFP excitation was performed with 488 nm light, and colocalization was observed using FM4-64 staining at a final concentration of 0.08% (v/ v), allowing for the visualization and photography of the mycelium.

### 2.5 Analysis of mycelium growth in mutants

Mycelial plugs (5 mm) from 5-day-old PDA plates were taken from the edge of the wild type, mutant, and complement strains, inoculated into the center of the PDA plates, and cultured at 28°C for 5 days. The morphology of the mycelium was observed under the microscope and photographed. After 7 days, the colony diameter was counted by the cross method. Then, 5 μL of safranine dye was dripped at approximately 1 cm from the edge of the wild type, mutant, and complement strains and placed at room temperature for 5 min. The penetration of safranin dye was observed and photographed. In addition, 1 × 10^6^ cfu/mL spore suspensions of wild-type, mutant, and complement strains were prepared, respectively. After shaking the culture for 16 h, the vacuole morphology was observed under a microscope and photographed. Each test was repeated three times, with three replicates each time.

### 2.6 Analysis of asexual reproduction of mutants

For conidial production, mycelial plugs (5 mm) from 5-day-old PDA plates were taken from the edge of the wild type, mutant, and complement strains, inoculated into PDB plates and the culture was shaken at 28°C for 5 days. Conidial suspension concentration was adjusted to 1 × 10^6^ conidia/mL and the culture was shaken at 28°C for 2 days. The amount of conidia was measured by collecting conidia from 2-day-old PDB cultures and confirming the concentration of the conidial suspensions using a hemocytometer.

For conidial germination rate, we incubated 1 × 10^6^ conidia/mL conidial suspensions at 28°C for 9 h. The rate of conidial germination was determined every 3 h. Each test was repeated three times. The conidial germination rate was calculated as follows: conidial germination rate (%) = (number of germinated conidia/total number of conidia) × 100.

### 2.7 Analysis of the sensitivity of the mutants to stress factors

To test sensitivity to infiltration, cell membrane, and cell wall, comparisons of wild-type, mutant, and complement strains were performed on PDA supplemented with 1.2 mol/L NaCl, 1.2 mol/L KCl, 0.05% SDS, and 0.05% Congo red. To test sensitivity to metal ions, comparisons of wild-type, mutant, and complement strains were performed on PDA supplemented with 0–500 mmol/L Ca^2+^, 3 mmol/L Mn^2+^, and 6 mmol/L Zn^2+^. To test the sensitivity to endoplasmic reticulum stress, comparisons of wild-type, mutant, and complement strains were performed on PDA supplemented with 0–12 mmol/L dithiothreitol (DTT).

The mycelial plugs (5 mm) from 5-day-old PDA plates were transferred to the different PDA solid media listed above. The plates were incubated at 28°C for 7 days. Each test was repeated three times, with three replicates each time. The growth inhibition rate was calculated as follows: growth inhibition rate (%) = [(Diameter on PDA-Diameter on PDA with stress) /Diameter on PDA] × 100.

### 2.8 Determination of ectoenzyme activity

Ectoenzyme activity was assayed using the culture filtrate from a 3-day-old PDA liquid culture.

The substrate ABTS was decomposed by laccase to produce ABTS free radical, and the absorbance coefficient at 420 nm was much larger than that of the substrate ABTS. The laccase activity can be calculated by measuring the rate of increase of ABTS free radicals (Song et al., 2010). The reaction mixture consisted of 30 mmol/L ABTS solution and the culture filtrate. The absorbance coefficient was evaluated at 420 nm after 20 min incubation at 60°C. The activity of laccase was determined through calculations based on the formula below.

Laccase activity (mmol/min/L) = (ΔA × V_1_ × 10^−3^) / (*ε* × d × V_2_ × T); ΔA: difference of absorbance; ε: molar extinction coefficient of ABTS, 36,000 L/mol/cm; d: optical path of 96-well plate; V_1_: total reaction volume; V_2_: sample reaction volume; T: reaction time.

3, 5-Dinitrosalicylic acid (DNS) and reducing sugar were reduced to brown-red amino compounds by co-heating. Within a certain range, the amount of reducing sugar is proportional to the color depth of the reaction solution. The activities of sucrase, glucoamylase, and cellulase can be calculated by measuring the absorbance coefficient at 540 nm. The substrate for glucoamylase activity was soluble starch. The culture filtrate was added to the substrate, and the reaction was carried out in a water bath at 40°C for 30 min. The substrate for sucrase activity was sucrose. The culture filtrate was added to the substrate, and the reaction was carried out in a water bath maintained at 25°C for 10 min. The substrate for cellulase activity was carboxymethyl cellulose. The culture filtrate was added to the substrate, the reaction was carried out in a water bath at 40°C for 30 min, and the mixture was immediately placed into a boiling water bath for 15 min to obtain the saccharified solution. Then, DNS was added and boiled for 10 min to complete the reaction (Miller, 1959). The absorbance coefficient was evaluated at 540 nm.

The activities of sucrase, glucoamylase, and cellulase (U/mL) = (x × V)/ (V × T); x = (ΔA + 0.0405)/1.429; ΔA: difference of absorbance; V: sample reaction volume; T: reaction time.

### 2.9 Infection assays

The virulence of the mutants was assayed on cabbage seedlings at the 2–3 true leaf stage. Conidial suspensions of wild-type, mutant, and complement strains were used as inocula, which was described above. The inoculum concentration was adjusted to 1 × 10^6^ conidia/mL, and the root-dip method was used in this study (Lv et al., 2011). Cabbage seedlings dipped in sterilized distilled water served as non-inoculated controls. Disease symptoms were assessed according to the disease severity every 3 days and divided into five levels according to pre-established rating scales (Li et al., 2015b). For each isolate, three independent biological replicates were carried out, and 24 seedlings were inoculated in each replicate.

## 3 Results

### 3.1 Identification of the Rab family proteins FoRab5, FoRab7, and FoRab8 in *Fusarium oxysporum*

The *FoRab5* gene encoded a 203 amino acid protein, the *FoRab7* gene encoded a 205 amino acid protein, and the *FoRab8* gene encoded a 238 amino acid protein, in which multiple Rab family motifs are found. Based on the prediction results of TMHMM, they are not transmembrane proteins. The conserved sequence motifs of the Ras superfamily, including G-boxes (G1–G5), Rab family motifs (RabF), and Rab subfamily motifs (RabSF), are present in the amino acid sequence of FoRab5, FoRab7, and FoRab8 (Supplementary Figure S1). There is a CXC motif at their carboxyl terminus. Phylogenetic analysis revealed that the amino acid sequences of FoRab5, FoRab7, and FoRab8 exhibited significant sequence similarity with the predicted orthologs from various other organisms (Supplementary Figure S2). This finding suggests that FoRab5, FoRab7, and FoRab8 are highly conserved throughout evolution.

### 3.2 Acquisition of gene deletion mutants and complements

The deletion mutants *ΔFoRab5*, *ΔFoRab7*, and *ΔFoRab8* were obtained by a target gene replacement through homologous recombination (Supplementary Figure S3). The regions flanking the target gene, including both the upstream and downstream sequences, as well as the selectable marker gene, were amplified. The process of homologous recombination, which involved these flanking regions and the replacement gene, resulted in the precise excision of the target gene and the complete substitution with the selectable marker gene. The mutants *ΔFoRab5*, *ΔFoRab7*, and *ΔFoRab8* were analyzed by PCR to confirm the correct deletion (Supplementary Figure S4). All mutants showed that the target gene was deleted and that the *hygR* gene was integrated into the correct position within the genome.

The complementary plasmids were introduced into *ΔFoRab5*, *ΔFoRab7*, and *ΔFoRab8* strains to construct complementary strains and verified by PCR (Supplementary Figure S5). All complementation strains were able to amplify the previously knocked-out gene fragments, indicating that the gene was successfully restored. All primer information used in this experiment is listed in Supplementary Table S1.

### 3.3 The subcellular localization results of the three proteins are different

The localization results predicted by the WoLF PSORT online platform showed that FoRab5 and FoRab8 may be localized in the cytoplasm, and FoRab7 may be localized in the vacuole. To obtain the accurate subcellular localization of FoRab5, FoRab7, and FoRab8 in Foc, we, respectively, constructed FoRab5-GFP, FoRab7-GFP, and FoRab8-GFP strains. The position of the green fluorescent protein was observed using a confocal laser scanning microscope, and the detection wavelength was 488 nm. FM4-64 was a membrane-selective red fluorescent dye that specifically binds to the plasma membrane and internal membrane organelles, emitting high-intensity fluorescence, and the detection wavelength was 543 nm. FM4-64 indicated the location of proteins within cells by co-localizing with GFP signals.

**Figure 1:**
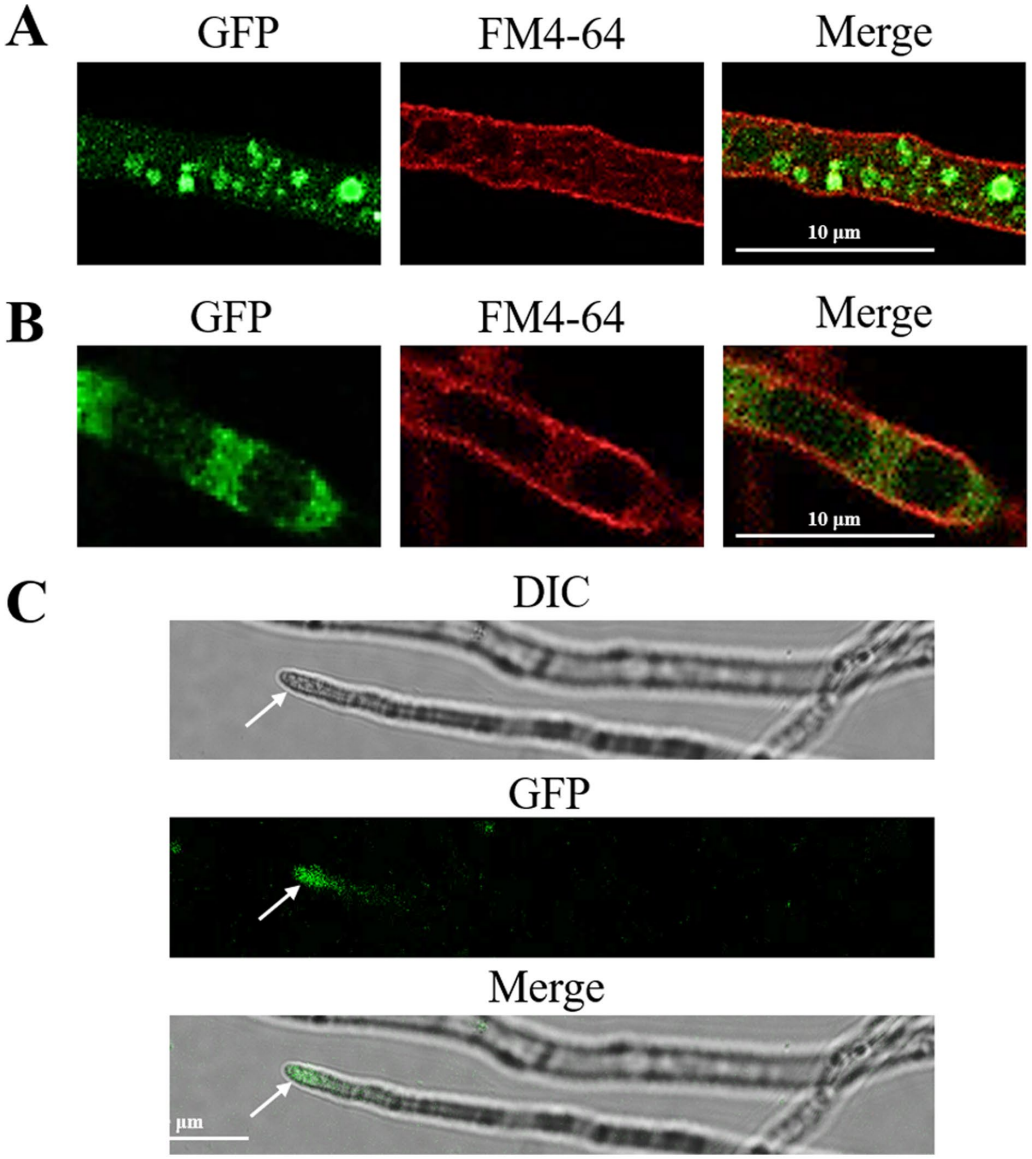
Subcellular localization of FoRab5, FoRab7, and FoRab8 GTPases. **(A)** Photomicrograph of subcellular localization of FoRab5. **(B)** Photomicrograph of subcellular localization of FoRab7. **(C)** Photomicrograph of subcellular localization of FoRab8. The confocal fluorescence images indicate that GFP fluorescence was detected in the mycelium of the GFP-FoRab5, GFP-FoRab7, and GFP-FoRab8 strains after 24 h of culture in the PDB medium, and these signals were found to co-localize with the FM4-64 marker.

In the FoRab5-GFP strain, following FM4-64 staining, the membrane system of the cells exhibited red fluorescence, while green fluorescence was observed as a punctate distribution in the cytoplasm. This suggests that the FoRab5 protein may be localized within vesicles of varying sizes (Figure 1A). This suggests that FoRab5 may be associated with the process of vesicle formation. Observations made using confocal microscopy revealed distinct circular green fluorescent signals on the vacuolar membrane of the FoRab7-GFP strain, which were found to co-localize with FM4-64 (Figure 1B). This suggests a potentially close relationship between FoRab7 and vacuolar function. In addition, the prominent green fluorescence signal was observed at the apex of the mycelium in the FoRab8-GFP strain (Figure 1C), suggesting that FoRab8 may play a role in the polar growth of the mycelium.

### 3.4 *ΔFoRab5*, *ΔFoRab7*, and *ΔFoRab8* mutants exhibit reduced radial growth and changes in colony morphology

**Figure 2:**
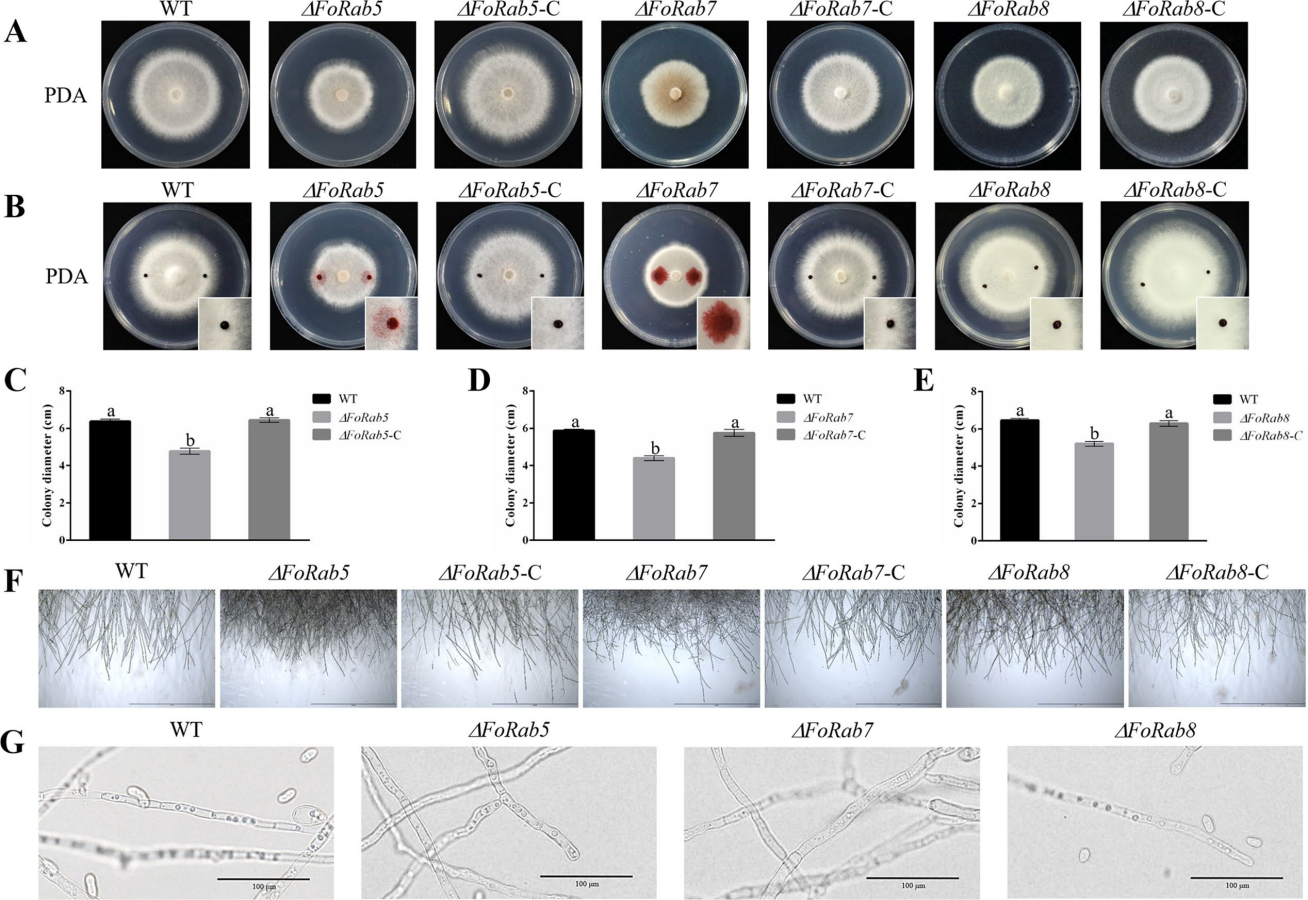
Investigating the role of *FoRab5*, *FoRab7*, and *FoRab8* in the growth of mycelium and vacuole morphology of Foc. **(A)** Colony morphology of WT, mutant, and complement strains on PDA medium. **(B)** Permeation of safranine dyes on the surface of WT, mutant, and complement mycelium in 5 min. **(C)** Growth diameter of WT, *ΔFoRab5*, and *ΔFoRab5-C* strains. **(D)** Growth diameter of WT, *ΔFoRab7*, and *ΔFoRab7-C* strains. **(E)** Growth diameter of WT, *ΔFoRab8*, and *ΔFoRab8-C* strains. **(F)** Observation of mycelial edge morphology of WT, mutant, and complement strains. **(G)** Observation of vacuole morphology in WT and mutant hyphae. Similar results were obtained in three independent biological replicates. The error bar represents the standard error of the mean. a, *p* > 0.05; b, *p* < 0.05.

Dramatic changes in vegetative growth were observed in the *ΔFoRab5*, *ΔFoRab7*, and *ΔFoRab8* mutants. The hydrophobicity of the mycelium was evaluated by the penetration of safranine dye. It was found that in the wild-type strain and the *ΔFoRab8* mutant, the dyes were attached to the surface of the mycelium; the dyes were partially infiltrated into the mycelium in the *ΔFoRab5* mutant; and they were completely infiltrated into the mycelium in the *ΔFoRab7* mutant (Figure 2B). The results showed that *FoRab5* and *FoRab7* were involved in the growth of aerial hyphae. Radial growth was evaluated as colony diameter on PDA plates. The growth rate of the three mutants was drastically reduced compared with the wild-type strain (Figures 2A,C–E). Compared to the wild-type strain, the hyphae at the edge of the colonies of the mutants *ΔFoRab5* and *ΔFoRab8* were denser, while the mutant *ΔFoRab7* had more branches (Figure 2F). In addition, when observing the morphology of the mycelium under a microscope, it was found that the vacuoles of the mutant *ΔFoRab7* were fragmented (Figure 2G). On that account, Rab7 may be closely related to the function of vacuoles.

### 3.5 *ΔFoRab5, ΔFoRab7*, and *ΔFoRab8* mutants showed a decrease in the number of conidia and a decrease in the spore germination rate

**Figure 3:**
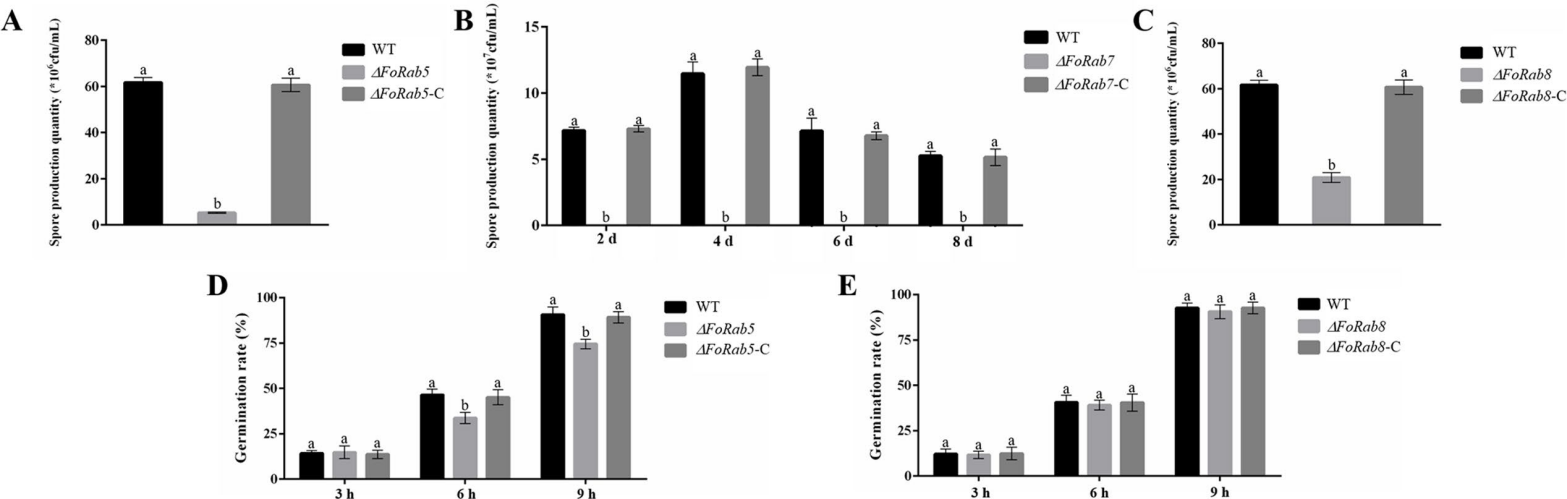
Investigating the role of *FoRab5*, *FoRab7*, and *FoRab8* in conidial production and germination of Foc. **(A)** The sporulation of WT, *ΔRab5*, and *ΔRab5*-C after 48 h of culture. **(B)** The sporulation of WT, *ΔRab7*, and *ΔRab7*-C every 2 days. **(C)** The sporulation of WT, *ΔRab8*, and *∆Rab8*-C after 48 h of culture. **(D)** The spore germination rate of WT, *ΔRab5*, and *ΔRab5*-C after 3, 6, and 9 h of culture. **(E)** The spore germination rate of WT, *Rab8*, and *∆Rab8*-C after culture for 3, 6, and 9 h. Similar results were obtained in three independent biological replicates. The error bar represents the standard error of the mean. a, *p* > 0.05; b, *p* < 0.05.

A significant decrease in the number of conidia was observed in the *ΔFoRab5*, *ΔFoRab7*, and *ΔFoRab8* mutants. Three mutant strains produced a significantly lower quantity of conidia when compared to wild-type and complemented strains. The wild-type strain produced 12 times more conidia than the *ΔFoRab5* mutant (Figure 3A). The *ΔFoRab7* mutant produced very few spores, which was 1,000 times less than the wild type (Figure 3B). The wild-type strain produced three times more conidia than did the *ΔFoRab8* mutant (Figure 3C). Because the sporulation of the *ΔFoRab7* mutant was not enough for the germination experiment, the difference in the spore germination rate between the other two mutants and the wild type was compared. The conidial germination rate of the 3-h mutant and the wild type did not show a significant difference when all strains were cultivated in PDB liquid culture, but the conidial germination rate of the 6-h and 9-h mutant *ΔFoRab5* was lower than that of the wild type (Figures 3D,E).

### 3.6 Response of *ΔFoRab5, ΔFoRab7*, and *ΔFoRab8* mutants to abiotic stress factors and different metal ion stresses

**Figure 4:**
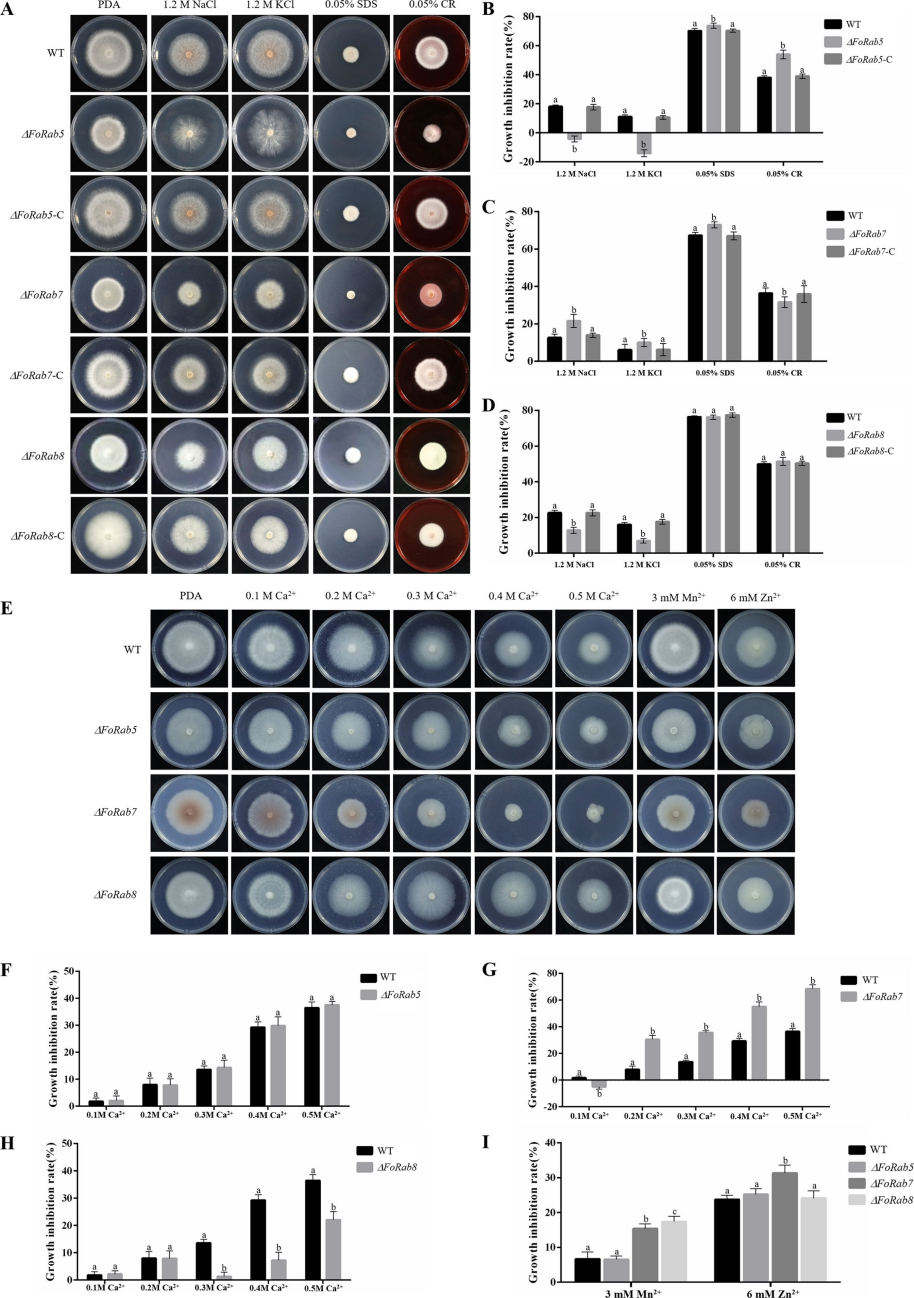
Exploring the roles of *FoRab5*, *FoRab7*, and *FoRab8* in Foc response to osmotic stress, cell membrane stress, cell wall stress, and metal ion stress. **(A)** Growth phenotypes of WT, mutant, and complement strains on PDA medium supplemented with 1.2 M KCl, 1.2 M NaCl, 0.05% SDS, and 0.05% Congo red. **(B)** Growth diameter of WT, *ΔFoRab5*, and *ΔFoRab5-C* strains shown in panel A. **(C)** Growth diameter of WT, *ΔFoRab7*, and *ΔFoRab7-C* strains shown in **(A)**. **(D)** Growth diameter of WT, *ΔFoRab8*, and *ΔFoRab8-C* strains shown in **(A)**. **(E)** Growth phenotypes of WT and mutant strains on PDA medium supplemented with 0.1–0.5 M Ca^2+^, 3 mM Mn^2+^, and 6 mM Zn^2+^. **(F)** Growth diameters of WT and *ΔFoRab5* strains under Ca^2+^ stress shown in **(E)**. **(G)** Growth diameters of WT and *ΔFoRab7* strains under Ca^2+^ stress shown in **(E)**. **(H)** Growth diameters of WT and *ΔFoRab8* strains under Ca^2+^ stress shown in **(E)**. **(I)** Growth diameters of WT, *ΔFoRab7*, and *ΔFoRab8* strains under Mn^2+^ and Zn^2+^ stress shown in **(E)**. Similar results were obtained in three independent biological replicates. The error bar represents the standard error of the mean. a, *p* > 0.05; b, *p* < 0.05.

To investigate whether the *FoRab5*, *FoRab7*, and *FoRab8* genes are involved in the response to environmental stress agents, we tested the vegetative growth of the strains on PDA medium amended with NaCl, KCl, SDS, and Congo red (Figure 4A). Compared to the wild-type strain, NaCl and KCl affected the colony morphology of the *ΔFoRab5* mutants, and SDS and CR significantly inhibited the growth rate (Figure 4B). NaCl, KCl, and SDS significantly inhibited the growth rate of the *ΔFoRab7* mutants, but CR inhibited the growth rate of the *ΔFoRab7* mutants less than that of the wild type (Figure 4C). The inhibitory effect of NaCl and KCl on the *ΔFoRab8* mutant was less than that of the wild type (Figure 4D).

To investigate whether the *FoRab5*, *FoRab7*, and *FoRab8* genes are involved in the response to metal ions, we tested the vegetative growth of the strains on PDA medium amended with Ca^2+^, Mn^2+^, and Zn^2+^ (Figure 4E). After adding Ca^2+^, the growth of the *ΔFoRab5*, *ΔFoRab7*, and *ΔFoRab8* mutants was inhibited. The difference was that the relative inhibition rate of the *ΔFoRab5* mutant was not significantly different from that of the wild type (Figure 4F), and the inhibition degree of the *ΔFoRab7* mutant was significantly higher than that of the wild type (Figure 4G). When the concentration of Ca^2+^ reached 300 mmol/L, the inhibition degree of the *ΔFoRab8* mutant began to be lower than that of the wild type (Figure 4H). After adding Mn^2+^, the mycelial growth of the *ΔFoRab7* and *ΔFoRab8* mutants was significantly inhibited compared to that of the wild type. However, after adding Zn^2+^, only the mycelial growth of the *ΔFoRab7* mutant was significantly inhibited (Figure 4I). Therefore, it is speculated that the *FoRab7* and *FoRab8* genes may regulate the transport of metal ions and affect cell osmotic pressure.

### 3.7 Response of *FoRab5*, *FoRab7*, and *FoRab8* genes to endoplasmic reticulum inhibition and their effects on ectoenzyme secretion

**Figure 5:**
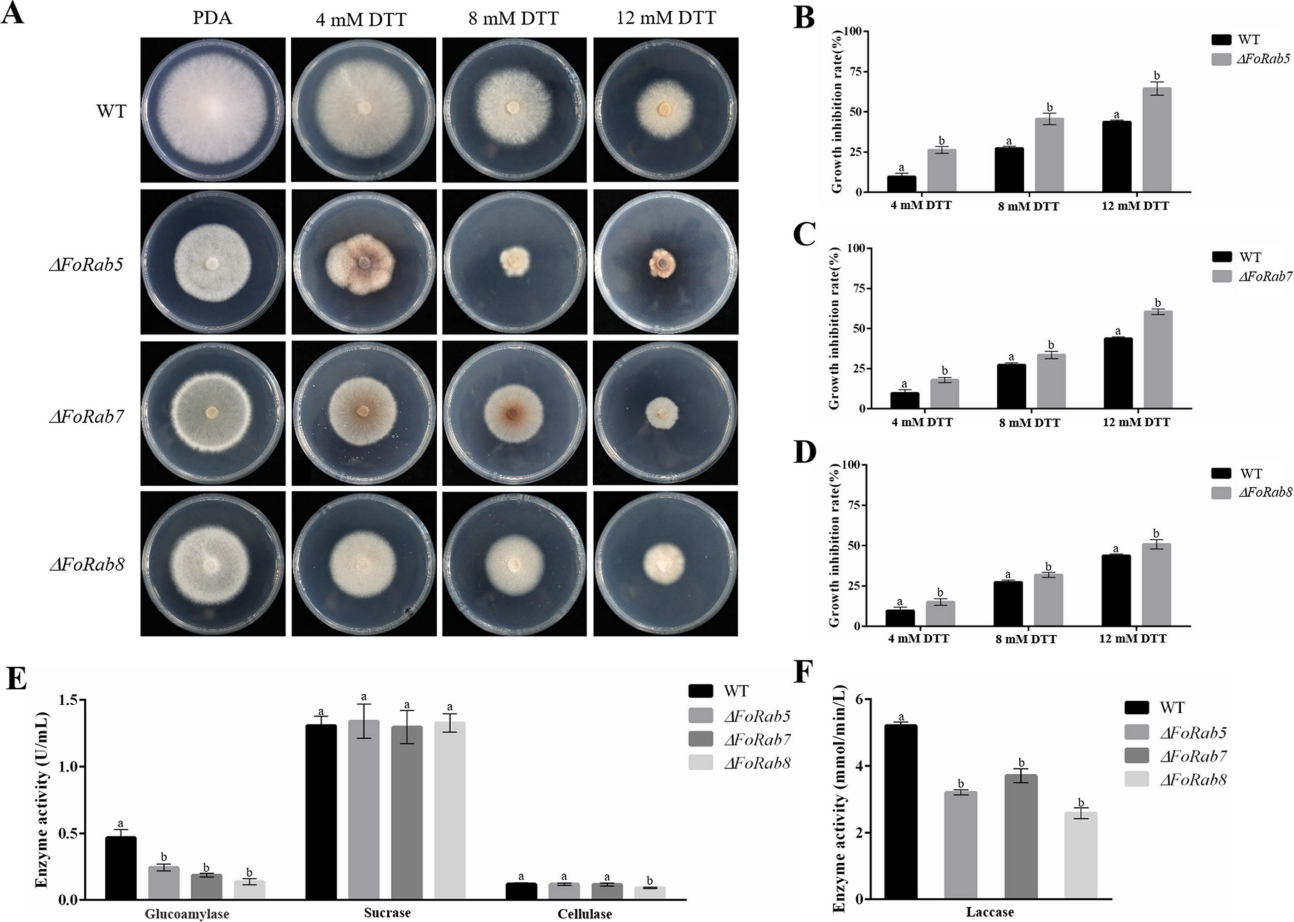
Effects of *FoRab5*, *FoRab7*, and *FoRab8* in Foc on endoplasmic reticulum inhibition and ectoenzyme secretion were determined. **(A)** Growth phenotypes of WT and mutant strains on PDA medium supplemented with 4 mM DTT, 8 mM DTT, and 12 mM DTT. **(B)** Growth diameters of WT and *ΔFoRab5* strains under DTT stress shown in panel A. **(C)** Growth diameters of WT and *ΔFoRab7* strains under DTT stress shown in **(A)**. **(D)** Growth diameters of WT and *ΔFoRab8* strains under DTT stress shown in **(A)**. **(E)** Comparison of glucoamylase, sucrase, and cellulase activities secreted by the *ΔFoRab5*, *ΔFoRab7*, and *ΔFoRab8* mutants. **(F)** Comparison of laccase activities secreted by the *ΔFoRab5*, *ΔFoRab7*, and *ΔFoRab8* mutants. Similar results were obtained in three independent biological replicates. The error bar represents the standard error of the mean. a, *p* > 0.05; b, *p* < 0.05.

To investigate whether the *FoRab5*, *FoRab7*, and *FoRab8* genes were involved in the response to endoplasmic reticulum inhibitors, we tested the vegetative growth of the strains on PDA medium amended with different concentrations of DTT (Figure 5A). After adding DTT, the mycelial growth of the *ΔFoRab5*, *ΔFoRab7*, and *ΔFoRab8* mutants was inhibited to varying degrees, and the degree of inhibition was significantly higher than that of the wild type (Figures 5B–D). Among them, the *ΔFoRab5* mutant had the highest degree of inhibition, and the vegetative growth of the mycelium was seriously damaged. Therefore, it is speculated that the *FoRab5*, *FoRab7*, and *FoRab8* genes may be involved in the regulation of endoplasmic reticulum protein transport, and the *FoRab5* gene may play a major regulatory role.

After the ectoenzyme matures, it is transported to the outside of the cell through vesicles to play its respective functions. In this experiment, laccase, glucoamylase, sucrase, and cellulase were selected for enzyme activity determination. The concentration of the product can be determined by measuring the change in absorbance of the solution before and after the reaction, which in turn allows for the calculation of enzyme activity. The experimental results indicate that the secretion of glucoamylase and laccase activities in the *ΔFoRab5*, *ΔFoRab7*, and *ΔFoRab8* mutants was significantly reduced compared to the wild type. Furthermore, the *ΔFoRab8* mutant also exhibited a notable decrease in cellulase activity (Figures 5E,F). This suggests that these three genes may be involved in the regulation of the secretion of ectoenzymes and that the genes regulating the secretion of various enzymes may differ. After the protein is synthesized on the endoplasmic reticulum ribosome, it is wrapped into the vesicle and folded by the Golgi. After the cell membrane is fused, the protein is released into the extracellular. Therefore, it is hypothesized that the reduction in ectoenzyme content in the mutants may be attributed to an obstruction in the intracellular vesicular transport process.

### 3.8 The pathogenicity of *ΔFoRab5, ΔFoRab7*, and *ΔFoRab8* mutants was significantly reduced

**Figure 6:**
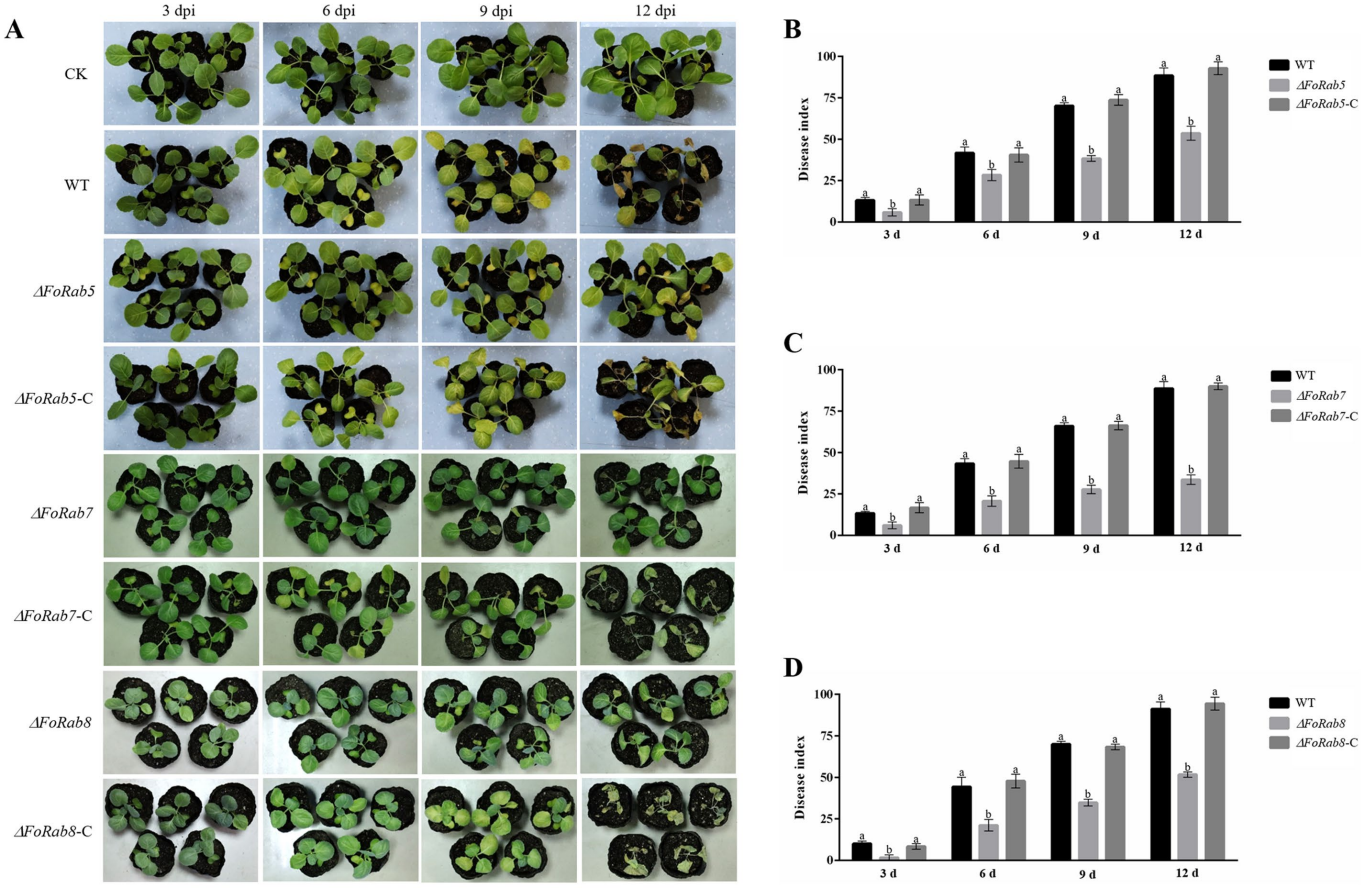
Effect of *FoRab5*, *FoRab7*, and *FoRab8* in Foc on the pathogenicity to cabbage seedlings. **(A)** The disease symptoms of cabbage seedlings inoculated with WT, mutant, and complement strains at 3, 6, 9, and 12 dpi. **(B)** Disease index of cabbage seedlings inoculated with WT, *ΔFoRab5*, and *ΔFoRab5-C* strains from 3 to 12 days after inoculation. **(C)** Disease index of cabbage seedlings inoculated with WT, *ΔFoRab7*, and *ΔFoRab7-C* strains from 3 to 12 days after inoculation. **(D)** Disease index of cabbage seedlings inoculated with WT, *ΔFoRab8*, and *ΔFoRab8-C* strains from 3 to 12 days after inoculation. CK represents cabbage seedlings treated with sterile water, and WT represents cabbage seedlings treated with WT strain spore solution. Each replicate included more than 30 seedlings for each isolate. Similar results were obtained in three independent biological replicates. The error bar represents the standard error of the mean. a, *p* > 0.05; b, *p* < 0.05.

To examine the role of the *FoRab5*, *FoRab7*, and *FoRab8* genes in cabbage infection, we compared the disease symptoms of the cabbage plants when inoculated with the wild-type strain and the *ΔFoRab5*, *ΔFoRab7*, and *ΔFoRab8* mutants. The disease index of the cabbage plants was counted every 3 days. During the whole disease cycle, cabbage plants inoculated with the wild-type strain began to turn yellow from the first true leaf to all leaves, and finally, the whole plant wilted and died. The cabbage seedlings inoculated with *ΔFoRab5*, *ΔFoRab7*, and *ΔFoRab8* mutants showed a significant reduction in the development of disease symptoms, and the yellowing degree of the leaves was significantly slowed down (Figure 6A). The pathogenicity was restored to the wild-type levels after gene complementation. By comparing the disease index of wild-type, mutant, and complement strains, we concluded that the *ΔFoRab5*, *ΔFoRab7,* and *ΔFoRab8* mutants had significantly reduced virulence in cabbage (Figures 6B–D). The results demonstrated that the *FoRab5*, *FoRab7*, and *FoRab8* genes play crucial roles in Foc that could influence its virulence in cabbage.

## 4 Discussion

Rab GTPases play important roles in many cellular processes, including membrane trafficking, cell growth, and cell differentiation. There are a limited number of reports on Rab GTPases in filamentous fungi. In this study, we identified and characterized *FoRab5*, *FoRab7*, and *FoRab8*, three genes encoding a vacuolar fusion protein in Foc. *FoRab5*, *FoRab7*, and *FoRab8* are required for vegetative growth, conidiogenesis, vesicle transport, and pathogenicity in cabbage.

Bioinformatic analysis was carried out on the whole genome of Foc (Fo5176^2^), and gene homology alignment was performed with *S. cerevisiae*. The homolog proteins FoRab5, FoRab7, and FoRab8 of Rab family proteins in Foc were analyzed and identified. Multiple sequence alignment analysis of the amino acid sequences showed that the amino acid sequences of these three proteins were highly homologous to the corresponding amino acid sequences from different races, indicating that Rab5, Rab7, and Rab8 proteins are highly conserved in evolution, and they may exercise similar functions in Foc. Of all the conserved motifs, the conservation within the G2 domain is particularly noteworthy as this region is the effector domain, responsible for functional specificity within the Rab GTPase family (Punt et al., 2001). Also contributing to Rab GTPase function are two conserved C-terminal cysteine residues that are posttranslationally modified to allow for, and stabilize, the protein’s association with vesicle membranes (Calero et al., 2003). In addition, the evolutionary relationship of the FoRab5, FoRab7, and FoRab8 proteins in Foc was analyzed. It was found that FoRab5, FoRab7, and FoRab8 of Foc were clustered in the same branch with *Fusarium*. However, the genetic relationship with *M. oryzae*, *Botrytis cinerea*, and *A. nidulans* gradually became more distant, while the genetic relationship with *S. cerevisiae* was the most distant. Interestingly, there are three Rab5 homologous proteins ScYpt51, ScYpt52, and ScYpt53 in *S. cerevisiae*, two Rab5 homologous proteins FgRab51 and FgRab52 in *F. graminearum*, two Rab homologous proteins, MoRab5A and MoRab5B in *M. oryzae*, and only one FoRab5 homologous protein in Foc. It has been observed that multiple Rab5 homologous proteins exhibit functional redundancy in *S. cerevisiae* and *F. graminearum* (Singer-Krüger et al., 1994; Zheng et al., 2015), and the functions of the two Rab5 homologous proteins in *M. oryzae* are independent (Qi et al., 2014). Therefore, it is speculated that FoRab5 in Foc may perform multiple functions.

Fungal hyphae are divided into vegetative hyphae and aerial hyphae according to their different parts and functions. The vegetative hyphae extend into the matrix to absorb essential nutrients, and the aerial hyphae will differentiate into various forms of sporulation structures after maturation. In a variety of pathogenic fungi, it was observed that Rab family proteins can regulate the vegetative growth of the mycelium. For instance, the deletion of the *Rab5* homologous gene *PsVPS21* in *Phytophthora sojae* resulted in a significant reduction in the growth rate of the *PsVPS21* deletion mutant, and the mycelium showed multi-branches, top and branch enlargement (Yao, 2014). The deletion of the *Rab7* homologous gene in *M. oryzae* led to a significant decrease in the aerial mycelium of the mutant, and the mycelial branches were curly (Chen et al., 2023). The deletion of the *Rab8* homologous gene *BcSas1* in *B. cinerea* also inhibited the growth rate of the mutant (Zhang et al., 2014). In this investigation, the mycelial growth rate of the *ΔFoRab5*, *ΔFoRab7*, and *ΔFoRab8* mutants on solid plates was significantly lower than that of the wild type. Further microscopic observation revealed that the number of mycelial branches of the three mutants was significantly higher than that of the wild type, indicating that these three genes may regulate the polar growth of the mycelium. In addition, the hydrophobicity of the hyphae in *ΔFoRab5* and *ΔFoRab7* mutants was significantly reduced, indicating that they may have affected the growth and development of hyphae.

The pathogenicity of Foc is an extremely complex process that consists of three parts: attachment and penetration of the infection structure (Kanani and Shukla, 2020; Li et al., 2013), cell wall-degrading enzymes, and toxins. Foc produces three asexual spores through asexual reproduction: large conidia, small conidia, and chlamydospores. Because the pathogen lacks the process of sexual reproduction, asexual spores play an important role in the infection cycle of Foc (Lu et al., 2023). The infection of host plants by Foc primarily occurs through conidia, and the germination of conidia is a pivotal initial step in the infection process. Therefore, the number of conidia produced by pathogens and the germination rate of conidia may be two of the important factors affecting the pathogenicity of pathogens. In this study, the conidial yields of the *ΔFoRab5*, *ΔFoRab7*, and *ΔFoRab8* knockout mutants were significantly lower than those of the wild type. In particular, in the *ΔFoRab7* mutant, almost no conidia were observed. Notably, the *ΔFoRab5* mutant also reduced the conidial germination rate. In addition, Foc causes disease by secreting a variety of toxins and cell wall-degrading enzymes, in which the secretion of cell wall-degrading enzymes is inseparable from vesicle transport (Gao et al., 2014). Foc secretes a series of cell wall-degrading enzymes during the root cell wall penetration until the colonization of the host plant, which plays a central role in the degradation of the plant cell wall and host–pathogen interaction (Caffall and Mohnen, 2009; Glass et al., 2013). In this study, the activity of cellulase was assessed. The findings revealed that the decomposition of sodium carboxymethyl cellulose in the *ΔFoRab8* mutant was significantly lower than that of the wild type, indicating that the cellulase activity of the mutant was significantly reduced. Therefore, it is speculated that the damage degree of the mutant *ΔFoRab8* to the cell wall of cabbage plants is lower than that of the wild type; that is, the pathogenicity of the mutant is reduced. Furthermore, the cellulase activity of the *ΔFoRab5* and *ΔFoRab7* mutants was not significantly different from that of the wild type, indicating that the pathogenicity of the pathogen is influenced by multiple factors. After that, Foc secretes a variety of mycotoxins during infection, which promotes infection and inhibits host plant defense against pathogens; the effects of these three Rab GTPases on toxins need to be further studied.

Each GTPase has a specific subcellular localization (Gotte et al., 2000). The subcellular localization of proteins is closely related to their biological function. GFP-FoRab5 is localized in vesicles and is mainly involved in endocytosis, which regulates the transport of vesicles from the cell membrane to the early endosome. The *ΔFoRab5* mutant is more sensitive to endoplasmic reticulum pressure, indicating that when the endoplasmic reticulum is inhibited by DTT, the synthesis and folding of proteins are affected, and the secretion pathway is hindered, resulting in a decrease in mycelial vegetative growth rate. GFP-FoRab7 is localized on the vacuolar membrane and is mainly involved in vacuolar fusion. The vacuolar membrane is an intracellular transporter responsible for regulating intracellular cation homeostasis (Ariño et al., 2010). Vacuoles can absorb and store excess heavy metal ions in the cytoplasm and isolate toxic heavy metal ions from other organelles to prevent their toxic effects. Due to the fragmentation of vacuoles after the deletion of *the FoRab7* gene, excess metal ions cannot be encapsulated by large vacuoles and dispersed in cells, resulting in an imbalance of cytoplasmic permeability and inhibition of mycelial growth. GFP-FoRab8 is localized in the acrosome of the mycelium and is mainly involved in the polar growth of the mycelium. The deletion of the *FoRab8* gene causes the transport of enzymes and components required for the decomposition and synthesis of the cell membrane and cell wall to be blocked, thus slowing down the growth rate of the mycelium.

## 5 Conclusion

In summary, the targeted deletion of *FoRab5*, *FoRab7*, and *FoRab8* had strong effects on growth, sporulation, pathogenicity, and ectoenzyme secretion in Foc. Future work will focus on the role of all Rab family proteins in the regulation of virulence in Foc.

## Data Availability

The datasets presented in this study can be found in online repositories. The names of the repository/repositories and accession number(s) can be found in the article/Supplementary material.

## Footnotes

^1^ https://www.genscript.com/wolf-psort.html

^2^ https://www.ncbi.nlm.nih.gov/datasets/genome/GCA_014154955.1
