# Supplementary material for: The small GTPases FoRab5, FoRab7, and FoRab8 regulate vesicle transport to modulate vegetative development and pathogenicity in Fusarium oxysporum f. sp. conglutinans
Source: Front Microbiol. 2025 Jan 29;16:1514092. doi: 10.3389/fmicb.2025.1514092 (PMC11814470; doi:10.3389/fmicb.2025.1514092)

1 Supplementary Figures and Tables

1.1 Supplementary Tables

Supplementary Table 1. Primers used in this study

Primer name	Primer sequence (5'-3')
Rab5-up-F	GTTGACGCTTGCGACCAGTC
Rab5-up-R	CATTGTTGACCTCCACTAGCTCCAGCCAAGCCCTCGGAGATGAGGTCAAG
Rab5-down-F	GGCAAAGGAATAGAGTAGATGCCGACCGGGGTCAAGTGGCGTATGGGTCG
Rab5-down-R	GATTGCAATCCTCGGCGGAC
Rab5-N-F	AGGAATTCGTTGCTCCGCTC
Rab5-N-R	GTGTTCGCAAACAGGACACG
Rab5-check-F	GCGCTTGGACAATCACATGC
Rab5-check-R	TGCCAACCAGTGCGATAACG
Rab7-up-F	CCAGTCTACTCCCCCATTGC
Rab7-up-R	CATTGTTGACCTCCACTAGCTCCAGCCAAGTTCTCGGGCTGTCTTCGACG
Rab7-down-2F	GGCAAAGGAATAGAGTAGATGCCGACCGGGGCTATCCTCGGTGGTGGATC
Rab7-down-R	TGTCCATGTCCGTGAGGAGC
Rab7-N-F	CCTTTTCAGGGGGAGTCAGC
Rab7-N-R	CTTAAGCATGCGGTCGATGG
Rab7-check-F	GATGATGGAAGCCACGCCAG
Rab7-check-R	AAGTTGGGAGGGTCCCTAGG
Rab8-up-F	ACCGAGGCTACCCAAGAGAC
Rab8-up-R	CATTGTTGACCTCCACTAGCTCCAGCCAAGGTGAGAGCAGCGGAAGAAGC
Rab8-down-F	GGCAAAGGAATAGAGTAGATGCCGACCGGGTCATTCTCCGTTTGCCGTCG
Rab8-down-R	CGATGAGAGCTGGGGAACTC
Rab8-N-F	ACCCAAGAGGGAAGCTCTGG
Rab8-N-R	TGGTGCGAATTCGAAGCCAG
Rab8-check-F	GTCAGGAGCGTTTCCGTACC
Rab8-check-R	TGTCTGCGGCTAGGCTGTAG
hygR-F	CTTGGCTGGAGCTAGTGGAGGT
hygR-R	CCCGGTCGGCATCTACTCTATTC
Rab5-SL-infu-F	TCGACGGTATCGATAAGCTTATGGCCGACTCTGCCAAC
Rab5-SL-infu-R	CCCCCGGGCTGCAGGAATTCGCAAGCACAGCTGTCCTTAG
Rab7-SL-infu-F	TCGACGGTATCGATAAGCTTATGTCTTCACGAAAGAAGGTCCTT
Rab7-SL-infu-R	CCCCCGGGCTGCAGGAATTCACAAGCGCAGCCATCACG
Rab8-SL-infu-F	TCGACGGTATCGATAAGCTTATGTCGAGTAATCGTAACTATGATT
Rab8-SL-infu-R	CCCCCGGGCTGCAGGAATTCGCAGCACTTGCTGCCGC

Supplementary Table 1 (Continue). Primers used in this study

Primer name	Primer sequence (5'-3')
pRGTN-F	TTACTGCGCACAAAGCCGAC
Rab5-SL-check-R	CCCAGTGCTTGGCCTTGATC
Rab7-SL-check-R	CAAGCACGACGAATGGGAAG
Rab8-SL-check-R	CGGTATCCCAGATCTGCAGC
neoR-check-F	CCAGGGTTTTCCCAGTCACG
neoR-check-R	GATCGACAAGACCGGCTTCC
Rab5-SL-H-check-F	CGTCGGAAAGTCGTCTCTCG
Rab5-SL-H-check-R	GCCTGAGCCTCTTCTGTTGG
Rab7-SL-H-check-F	CCTAACTCGAGAGGTCCTCG
Rab7-SL-H-check-R	TATCGCCCTTGGACTGGCAG
Rab8-SL-H-check-F	TCTGCTGCGATTCAGCGAAG
Rab8-SL-H-check-R	GGCTGTAGAAGGCCTTGTCG

1.2 Supplementary Figures

Supplementary Figure 1. The conserved domain analysis of FoRab5, FoRab7 and FoRab8 GTPases.

(A) Conserved domain analysis of FoRab5 GTPase. (B) Conserved domain analysis of FoRab7 GTPase. (C) Conserved domain analysis of FoRab8 GTPase. The multiple alignment of amino acid sequences were performed using ESPript 3.0, and their conserved domains were labeled with different colors. Five conserved domains G1-G5 and C-terminal red box with membrane localization signal, Rab family specific amino acid motif RabFl-RabF5 marked blue, Rab subfamily specific amino acid motif RabSFl-RabSF4 marked green.

A

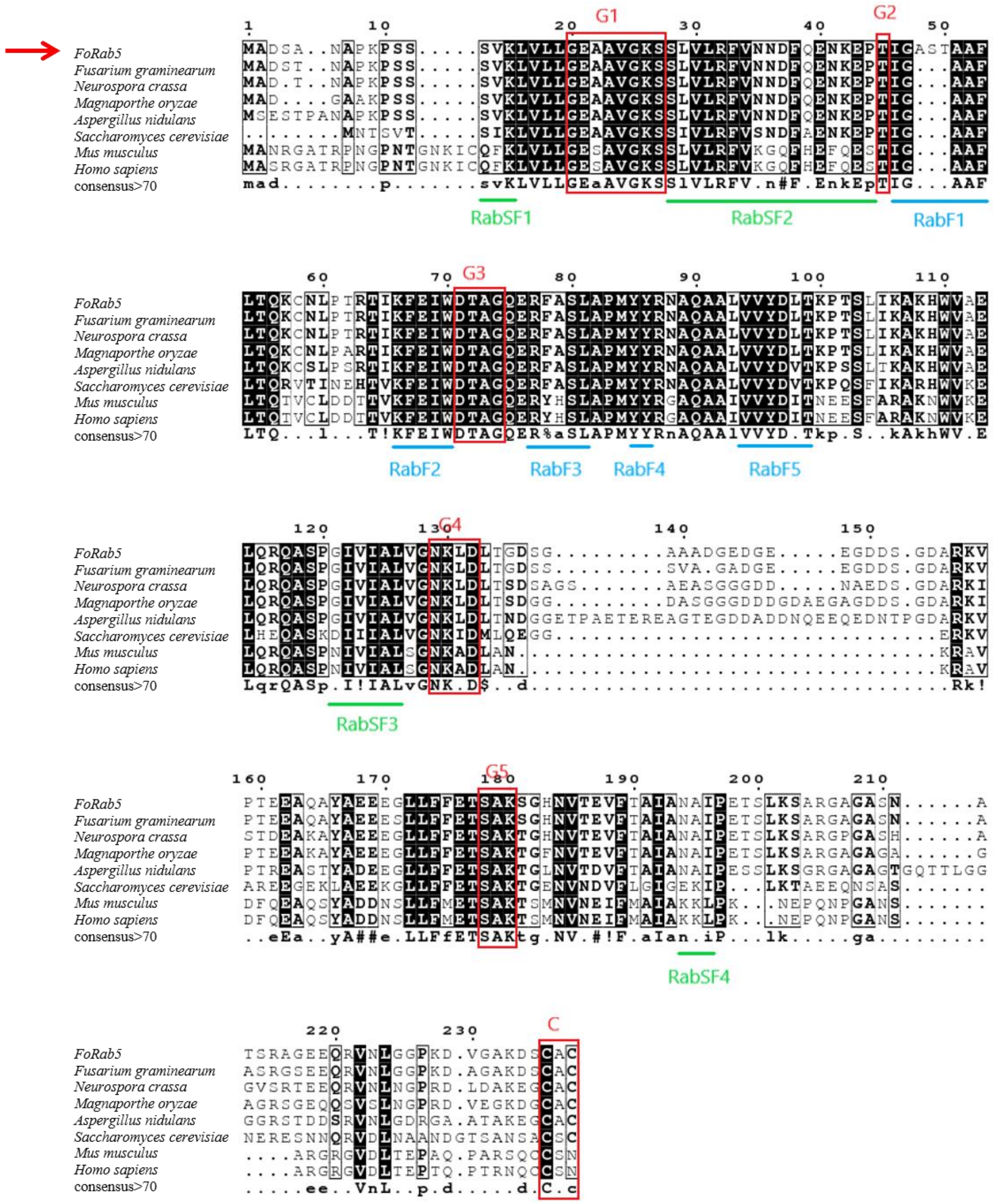
FoRab5
Fusarium graminearum
Neurospora crassa
Magnaporthe oryzae
Aspergillus nidulans
Saccharomyces cerevisiae
Mus musculus
Homo sapiens
consensus>70
G1
G2
G3
G4
G5
C
RabSF1
RabSF2
RabSF3
RabSF4
RabF1
RabF2
RabF3
RabF4
RabF5

B

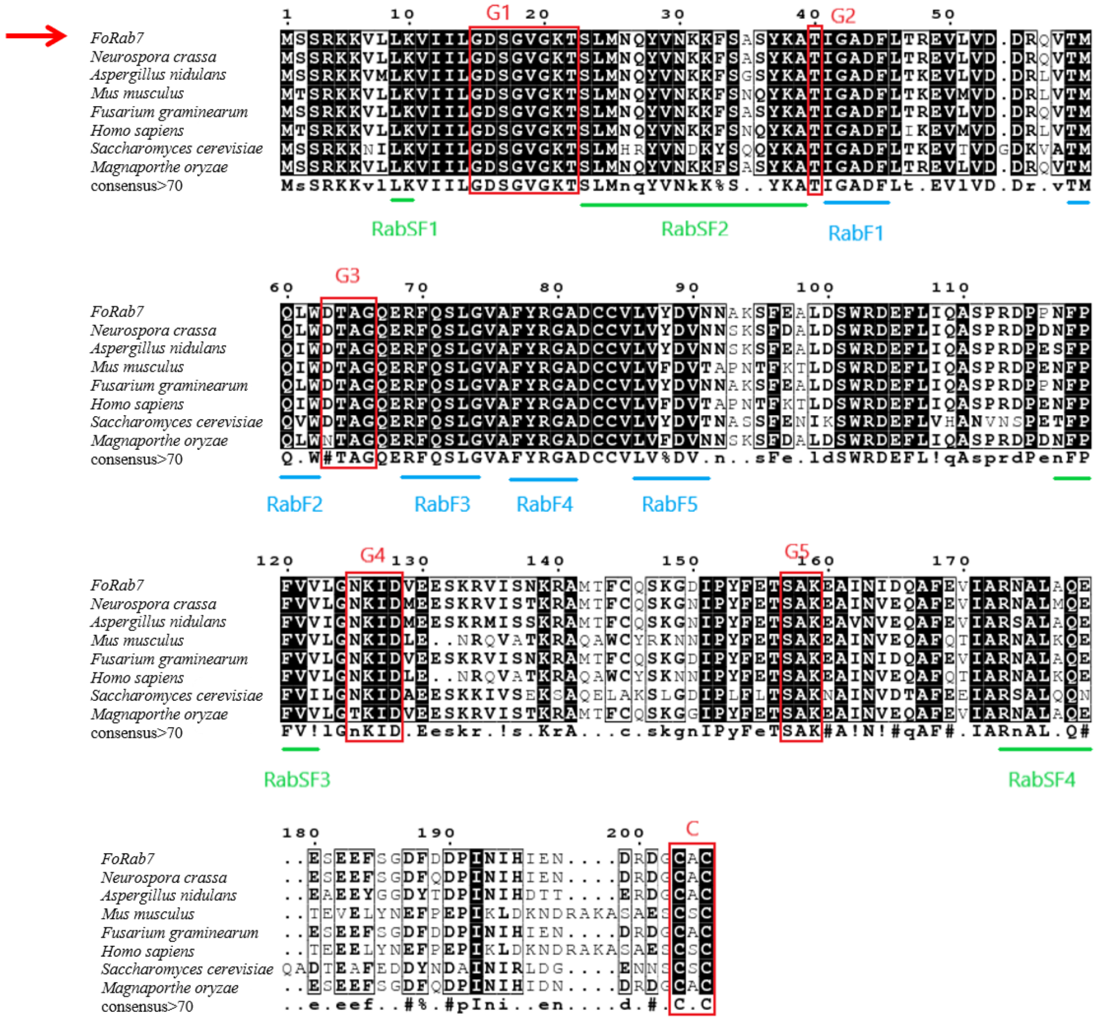
G1
G2
G3
G4
G5
C
FoRab7
Neurospora crassa
Aspergillus nidulans
Mus musculus
Fusarium graminearum
Homo sapiens
Saccharomyces cerevisiae
Magnaporthe oryzae
consensus>70
RabSF1
RabSF2
RabF1
RabF2
RabF3
RabF4
RabF5
RabSF3
RabSF4

C

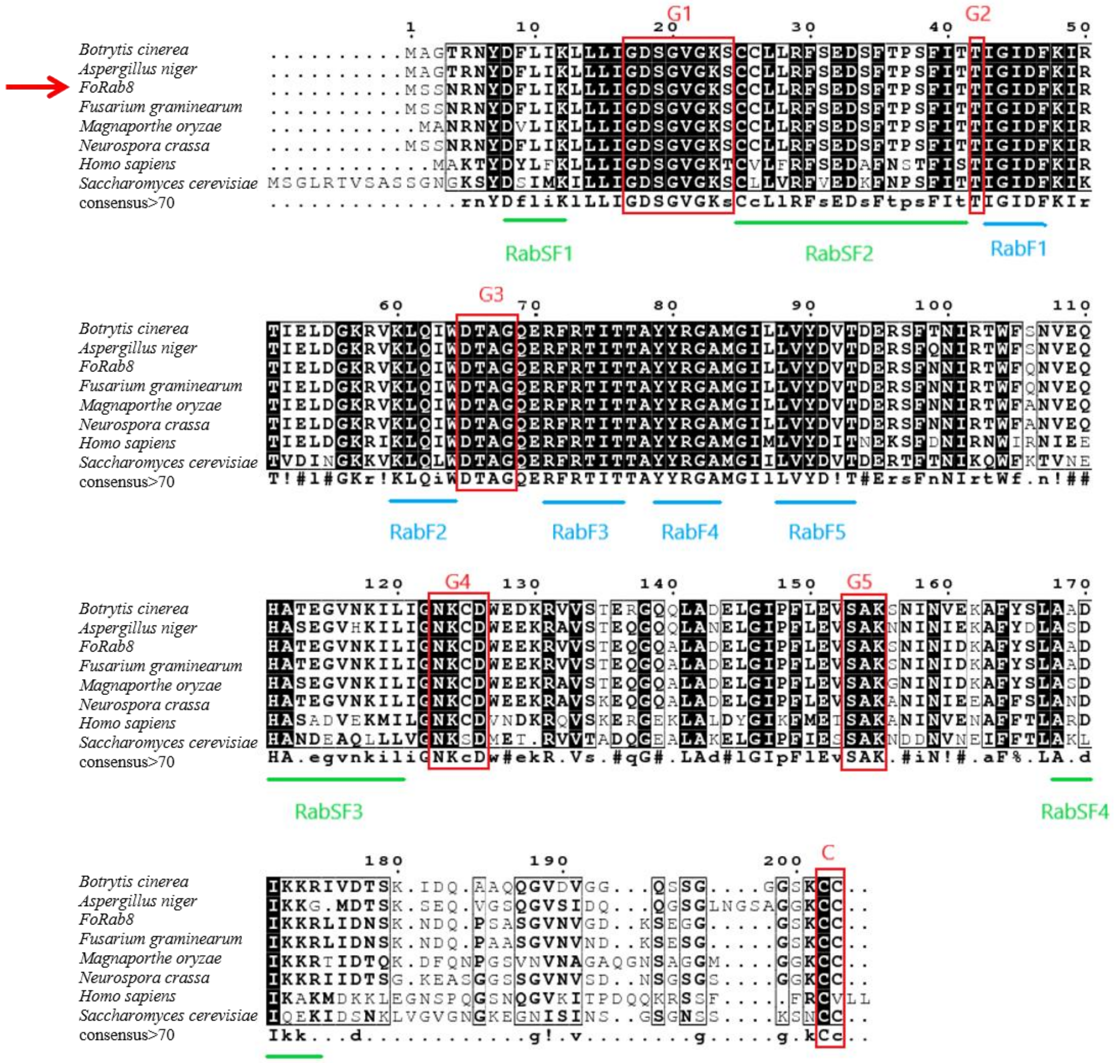

G1
G2
G3
G4
G5
C
Botrytis cinerea
Aspergillus niger
FoRab8
Fusarium graminearum
Magnaporthe oryzae
Neurospora crassa
Homo sapiens
Saccharomyces cerevisiae
consensus>70
RabSF1
RabSF2
RabSF3
RabSF4
RabF1
RabF2
RabF3
RabF4
RabF5

Supplementary Figure 2. The phylogenetic tree of FoRab5, FoRab7 and FoRab8 GTPases.

(A) Phylogenetic tree of FoRab5 GTPase. (B) Phylogenetic tree of FoRab7 GTPase. (C) Phylogenetic tree of FoRab8 GTPase. The phylogenetic trees were constructed based on the maximum likelihood method using MAGE7.

A

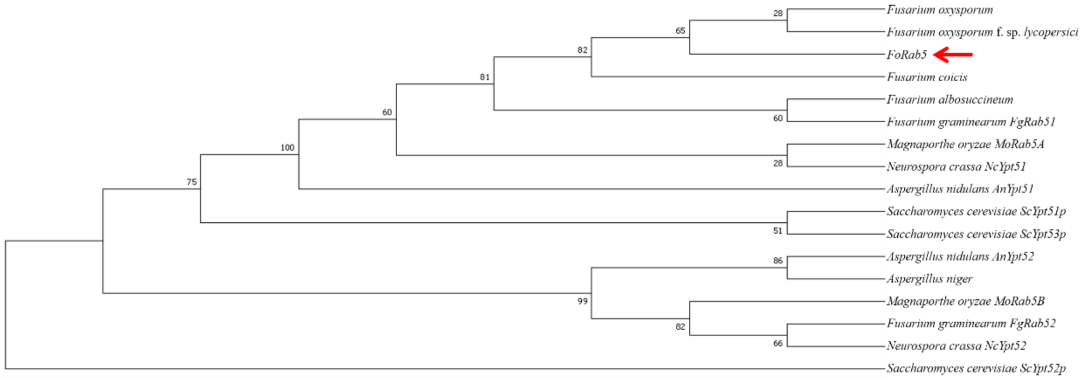

B

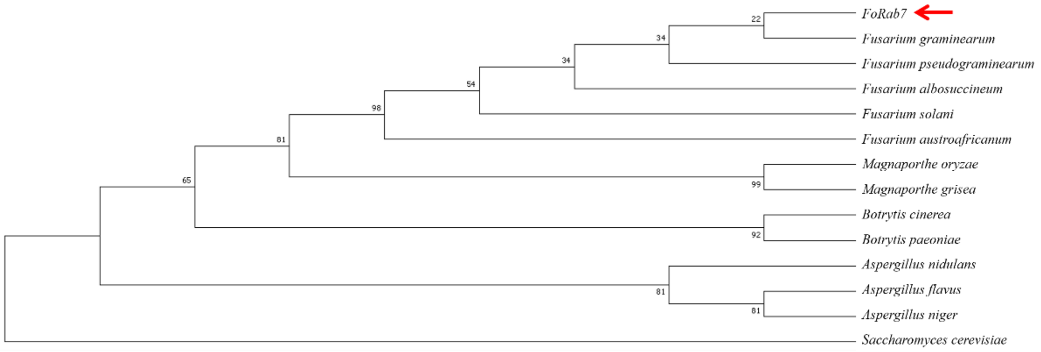

C

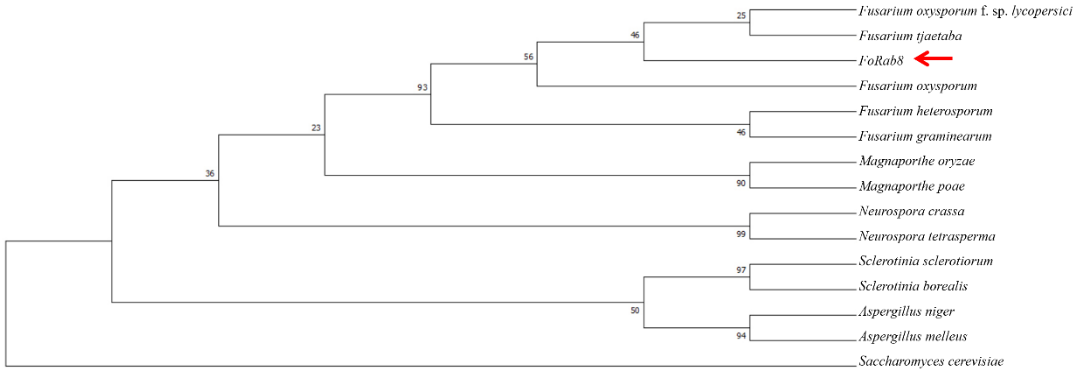

Supplementary Figure 3. Schematic diagram of homologous replacement arms.

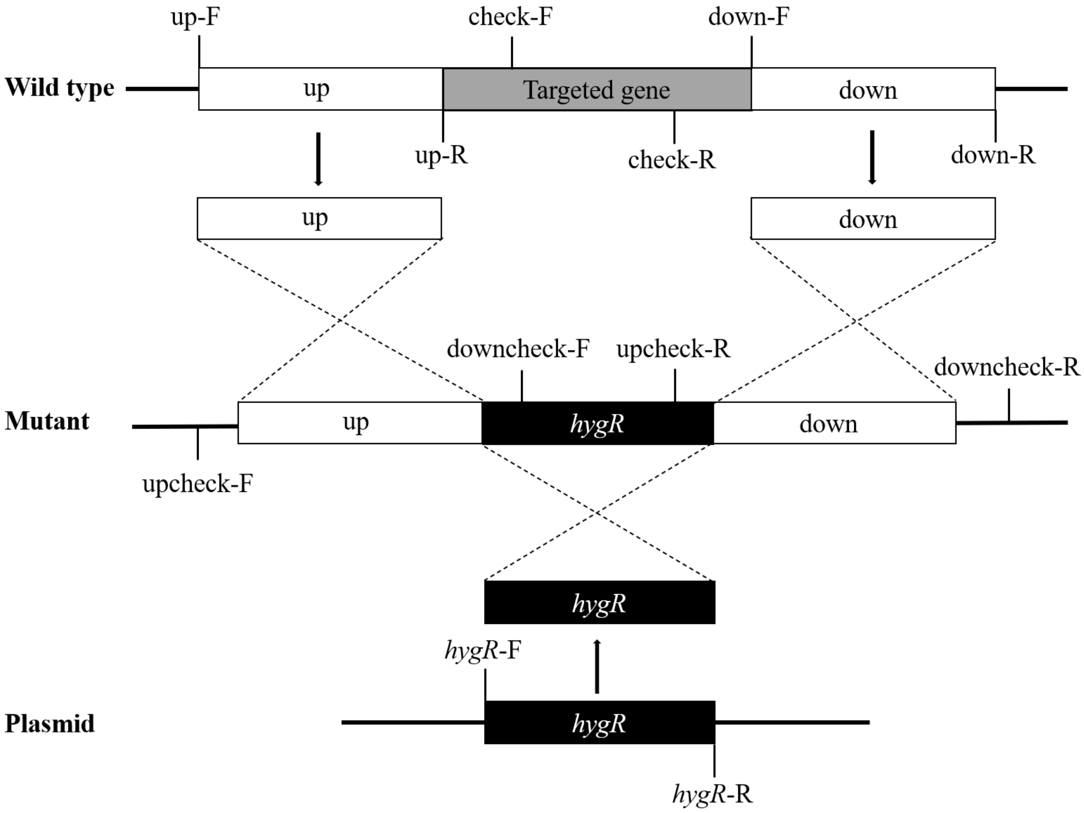

Supplementary Figure 4. PCR verification of *ΔFoRab5*, *ΔFoRab7* and *ΔFoRab8* deletion mutants.

(A) Electrophoretogram of *ΔFoRab5* deletion mutant identified by PCR. (B) Electrophoretogram of *ΔFoRab7* deletion mutant identified by PCR. (C) Electrophoretogram of *ΔFoRab8* deletion mutant identified by PCR. Among them, M is Trans 2K Plus II DNA Maker, 1 in the figure represents the wild type strain, and 2 represents the knockout mutant strain.

A

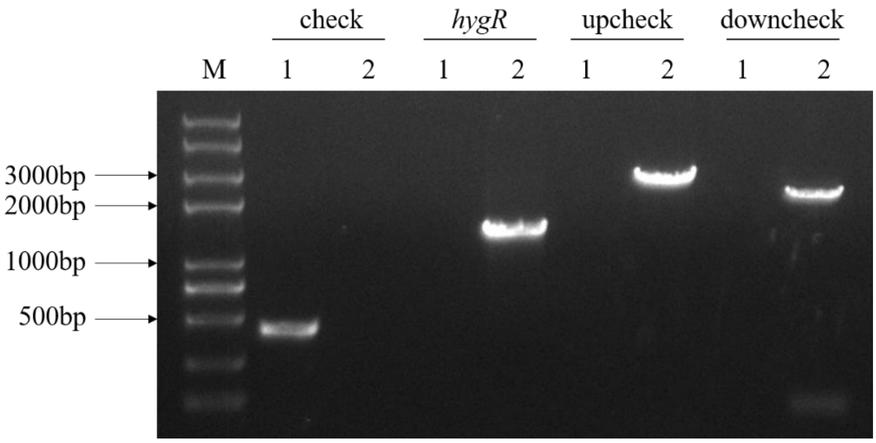

B

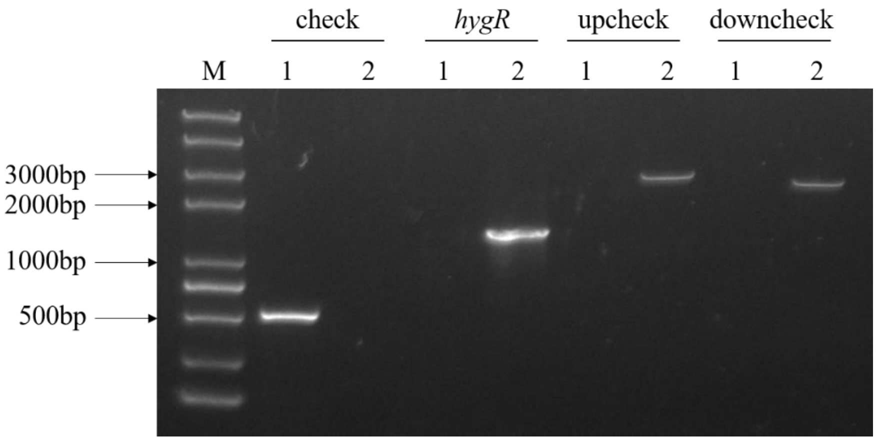

C

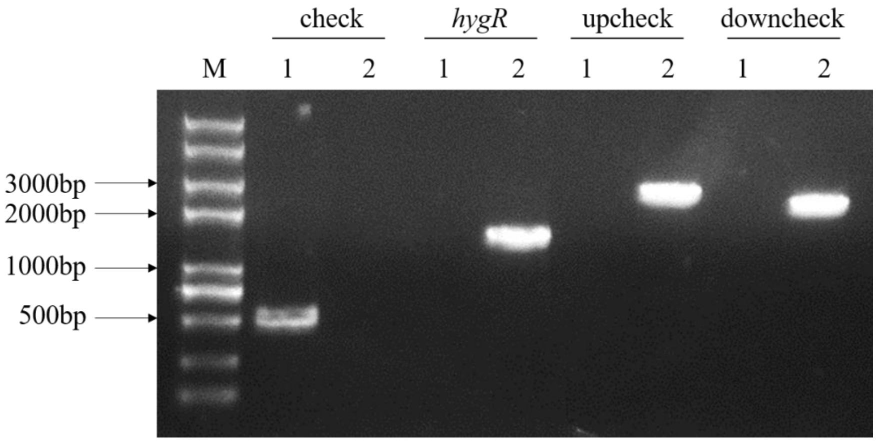

Supplementary Figure 5. PCR verification of *ΔFoRab5*-C, *ΔFoRab7*-C and *ΔFoRab8*-C complements.

(A) Electrophoretogram of *ΔFoRab5*-C complement identified by PCR. (B) Electrophoretogram of *ΔFoRab7*-C complement identified by PCR. (C) Electrophoretogram of *ΔFoRab8*-C complement identified by PCR. Among them, M is Trans 2K Plus DNA Maker, 1 in the figure represents the wild-type strain, 2 represents the knockout mutant strain, and 3 represents the complement strain.

A

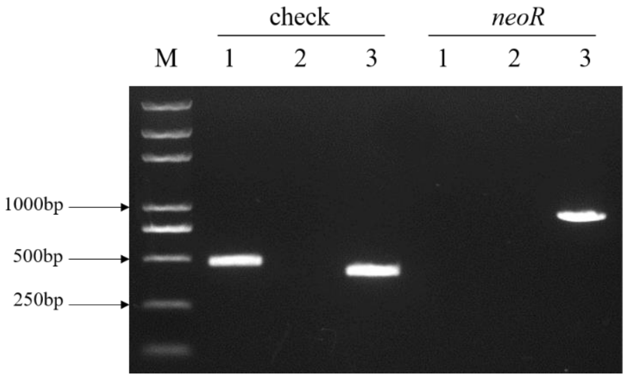

B

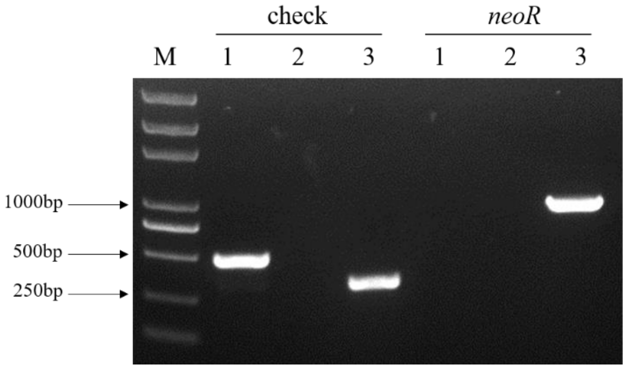

C

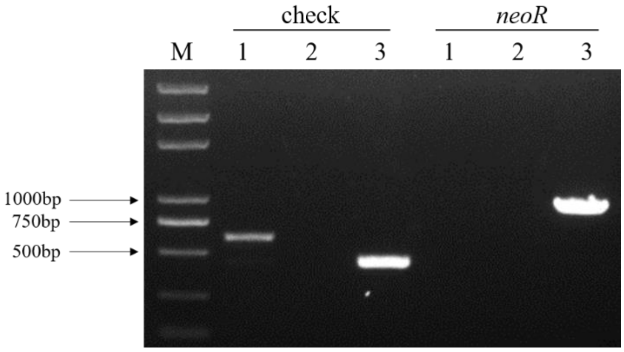

Supplement: Supplementary file 1 [file Data_Sheet_1.PDF]
